# Artificial intelligence in radiology

**DOI:** 10.1016/j.ejro.2024.100547

**Published:** 2024-01-06

**Authors:** Lorenzo Faggioni, Francesca Coppola

**Affiliations:** a University of Pisa; b University of Bologna and Faenza Hospital

Artificial intelligence (AI) has paved the way for the development of a wide range of new applications in medicine, harnessing the computational power of modern IT infrastructure for tackling tasks that allow delivering better patient care.

Radiologists have been at the forefront of the so-called 'AI revolution' since its beginnings, in view of the pivotal role of radiology in the diagnostic and therapeutic workup of many disease conditions and of the wealth of information stored (and largely hidden) inside medical images. Several radiology applications of AI have come under the spotlight, including AI systems for early disease detection, noninvasive extraction of novel biomarkers, individualised treatment planning and outcome prediction, and workflow optimisation in radiology departments, just to mention some.

While it is impossible to condense such a broad and rapidly evolving field in a limited space, we are happy that this Special Issue has finally taken quite a multifaceted shape, covering a variety of topics related to the use of AI in radiology. It contains several research articles on AI and multimodal musculoskeletal, chest, emergency and oncologic imaging, some of which are based on radiomics, an advanced and intriguing topic tightly connected with AI.

We wish to thank all authors for their valuable contributions and their choice to join this Special Issue as a means to disseminate their research within the medical community.

